# A Trade‐Off Between Leaf Carbon Economics and Plant Size Among Mangrove Species in Dongzhaigang, China

**DOI:** 10.1002/ece3.70559

**Published:** 2024-11-19

**Authors:** Dalong Jiang, Tao Nie, Qiuyu He, Jing Yan, Erhui Feng, Qing Ye

**Affiliations:** ^1^ Guangdong Provincial Key Laboratory of Applied Botany, and Key Laboratory of National Forestry and Grassland Administration on Plant Conservation and Utilization in Southern China, South China Botanical Garden Chinese Academy of Sciences Guangzhou China; ^2^ Ministry of Education Key Laboratory for Ecology of Tropical Islands, Key Laboratory of Tropical Animal and Plant Ecology of Hainan Province College of Life Sciences, Hainan Normal University Haikou China; ^3^ Hainan Dongzhaigang Mangrove Ecosystem Provincial Observation and Research Station Haikou China; ^4^ Hainan Dongzhaigang National Nature Reserve Authority Haikou Hainan China; ^5^ College of Life Sciences, Gannan Normal University Ganzhou China

**Keywords:** coastal forest, growth form, height and diameter, intertidal zone, resource strategies

## Abstract

Plant size is closely linked to its leaf trait characteristics, which are essential for determining its form and function. These relationships constitute a fundamental component of the global spectrum of plant diversity. Despite this, the size–trait relationships in coastal mangroves have often been overlooked, with a common assumption that they would mirror those found in terrestrial tropical trees. However, recent studies have begun to challenge this assumption, revealing unique adaptations and trait variations in mangroves that are influenced by their specific environmental conditions, such as salinity and nutrient availability. In this research, we investigated the leaf structural traits, plant height, and diameter at breast height or basal height (DBH) of 10 shrub and tree species. This study was carried out along an intertidal gradient within a mangrove forest located in Southeast China. We found that leaf traits differed significantly between shrubs and trees in their response to intertidal gradients, indicating that different species have evolved specific adaptations to thrive in their respective intertidal zones. This insight can help us decipher the selective pressures that have shaped trait evolution. Among all species, leaf carbon (C) economics (leaf dry mass content, leaf mass per area, and leaf density) decreased significantly with increasing plant height and DBH. For each growth form and intertidal zone, the relationships between plant size (height or DBH) and leaf C economics traits were consistent with those in the pooled dataset. Our study reveals that mangrove plants exhibit size‐related adjustments in leaf C economic strategies, indicating that plant size potentially acts as a proxy for the “slow–fast” continuum of plant performance. This discovery is pivotal for advancing our understanding of plant functional ecology and for enhancing the precision of global C cycle models, which are highly responsive to perturbations in atmospheric CO_2_ and climate change.

## Graphical abstract

Shrubs and trees show distinct leaf traits in response to intertidal gradients. Larger mangrove plants have lower leaf carbon efficiency. For each growth form and intertidal zone, plant size–trait patterns remain applicable.

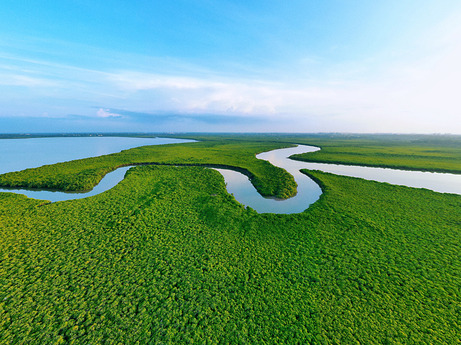

## 1 Introduction

Leaf functional traits are effective indicators of the ecological strategies employed by species and their adaptive performance within a specific environmental context (Iida et al. 2014; Asao et al. 2020; Mueller, Kray, and Blumenthal 2024). They have the potential to encapsulate plant strategies that pertain to water‐use efficiency, growth dynamics, and nutrient acquisition (Roskilly et al. 2019; Visakorpi et al. 2023). For example, an increase in the leaf mass per area (LMA) and leaf thickness (LT) indicates greater investments in leaf carbon (C) structures and a longer leaf lifespan, which, in turn, enhance the mean nutrient residence time in leaves (Wright et al. 2004; Díaz et al. 2016). Understanding leaf trait variation is crucial for delineating niche differentiation, elucidating competitive exclusion dynamics, and interpreting the mechanisms of community assembly (Valverde‐Barrantes et al. 2017; Bergmann et al. 2020). Nevertheless, the relationships between leaf functional traits and plant size remain unclear.

The leaf economics spectrum (LES) represents a well‐established framework within the realm of plant functional ecology that describes trait covariation relevant to C and nutrient economics across plant species (Wright et al. 2004; Mueller, Kray, and Blumenthal 2024). It has been reported that less costly structural leaf phenotypes, such as low LMA and low leaf dry matter content (LDMC), are commonly linked to a suite of traits that enhance rapid growth and resource acquisition, including elevated leaf nutrient concentrations and increased metabolic rates (Guimarães, dos Santos, and Ferreira 2022; Yan et al. 2023). Conversely, the opposite traits (high LMA and LDMC) are associated with conservative economics, which are slower growth rates, reduced resource uptake, and decreased tissue turnover (Joswig et al. 2022). Considering that leaf fall in mangrove species such as *Avicennia germinans* and *Rhizophora mangle* is more pronounced during the year's driest season, an increase in LMA and the investment in carbon‐intensive compounds like lignin or lipids would increase the developmental costs of leaves (Méndez‐Alonzo, López‐Portillo, and Rivera‐Monroy 2008). However, this trade‐off could simultaneously lead to more efficient water use by reducing the leaf's photosynthetic area, which, in turn, could increase the leaf longevity (del Campo et al. 2022).

Leaf economics and plant size (plant height and diaspore mass) represent two pivotal dimensions—exemplifying a decoupled correlation—that are fundamental to life‐history strategies across the global spectrum of plant form and function (Díaz et al. 2016; He et al. 2024). However, the findings differ among studies, and the field is far from resolved (Dayrell et al. 2018; Park et al. 2019; Zheng et al. 2022). Several studies have related the traits of LES (e.g., leaf area, LA; specific leaf area, SLA; and leaf nitrogen concentration, LNC) to growth rates (dos Santos and Ferreira 2020; Simpson et al. 2020). In principle, since tree size affects access to resources and, thereby, growth rates (Piponiot et al. 2022), it is expected that tree size is associated with leaf economic traits (Iida et al. 2014; He and Yan 2018). For example, larger trees tend to preempt light resources to smaller trees that, in turn, enables faster growth among trees of larger stature (Maynard et al. 2022). Previous studies have shown that LA and LNC increased among larger plants (He and Yan 2018; Zheng et al. 2022), which is interpreted as the result of plants adopting acquisitive economic strategies in response to higher growth rates through acclimation and plasticity. However, larger trees exhibit heightened vulnerability to environmental stressors such as drought and higher solar irradiance (Rozendaal, Hurtado, and Poorter 2006; Bennett et al. 2015; McGregor et al. 2021). Consequently, leaf traits often undergo corresponding shifts toward more conservative economic strategies as plant size increases, as exemplified by reductions in SLA and LA, along with an increase in LDMC (Kenzo et al. 2015; Dayrell et al. 2018; Park et al. 2019). Therefore, the effects of plant size on leaf economics are not consistent across studies.

Numerous recent studies have explored the relationships between plant size encompassing plant height and diameter at breast height and leaf traits in terrestrial plants (Park et al. 2019; Thomas et al. 2020; Zheng et al. 2022). Mangroves, having evolved from terrestrial flowering plants to colonize the coastal margins, have developed specialized morphological structures and physiological and ecological traits to thrive in the unique intertidal zone environment (Yu et al. 2023). This environment is marked by challenges such as high salinity, strong winds, high temperatures, siltation, and low oxygen content, as discussed by Friess et al. (2019). However, plant size and leaf trait relationships have not been well studied in coastal mangroves, possibly with the assumption that mangroves would exhibit similar leaf trait values and size–trait patterns to those of terrestrial tropical trees.

Mangroves constitute an ecological assemblage of trees and shrubs that have adapted to thrive in the intertidal zones of tropical and subtropical coastal regions. The intertidal zone experiences considerable fluctuations in moisture and temperature between the highest tides, when it is submerged, and the lowest tides, when it is exposed to air and sun (Weitzman et al. 2021). This zone is distinguished by a gradient that ranges from high to low and is influenced by the continental shelf's structure, variations in tidal fluctuations, and the succession of plant communities (Yu et al. 2023). The interplay of sediment formation matrices, sedimentation rates, and the extent and duration of tidal waterlogging among intertidal zones leads to a diverse array of sediment characteristics, including nutrient composition, salinity, oxygen levels, and temperature (Hayes et al. 2017; Ma et al. 2020). Considering that salinity and temperature are paramount environmental factors influencing mangrove functional traits (Medina‐Calderón et al. 2021; Lang et al. 2022), mangroves could have specialized structural traits along intertidal gradients (Yu et al. 2023) and thus provide a unique opportunity to improve our understanding of plant size–trait relationships.

In this study, we examined leaf traits and their relationships with plant size in a sample of 10 dominant mangrove species in Dongzhaigang, China. We hypothesize that (1) leaf functional traits exhibit a significant variation across growth forms and intertidal zones due to the differential responses of various growth forms to changing environmental conditions along the intertidal gradient (Islam et al. 2024) and (2) smaller species are inclined to adopt increasingly conservative economic strategies characterized by high LD, LMA, and LDMC, as they are more susceptible to C starvation induced by shading (McDowell et al. 2018).

## 2 Materials and Methods

### 2.1 Site Description

**FIGURE 1:**
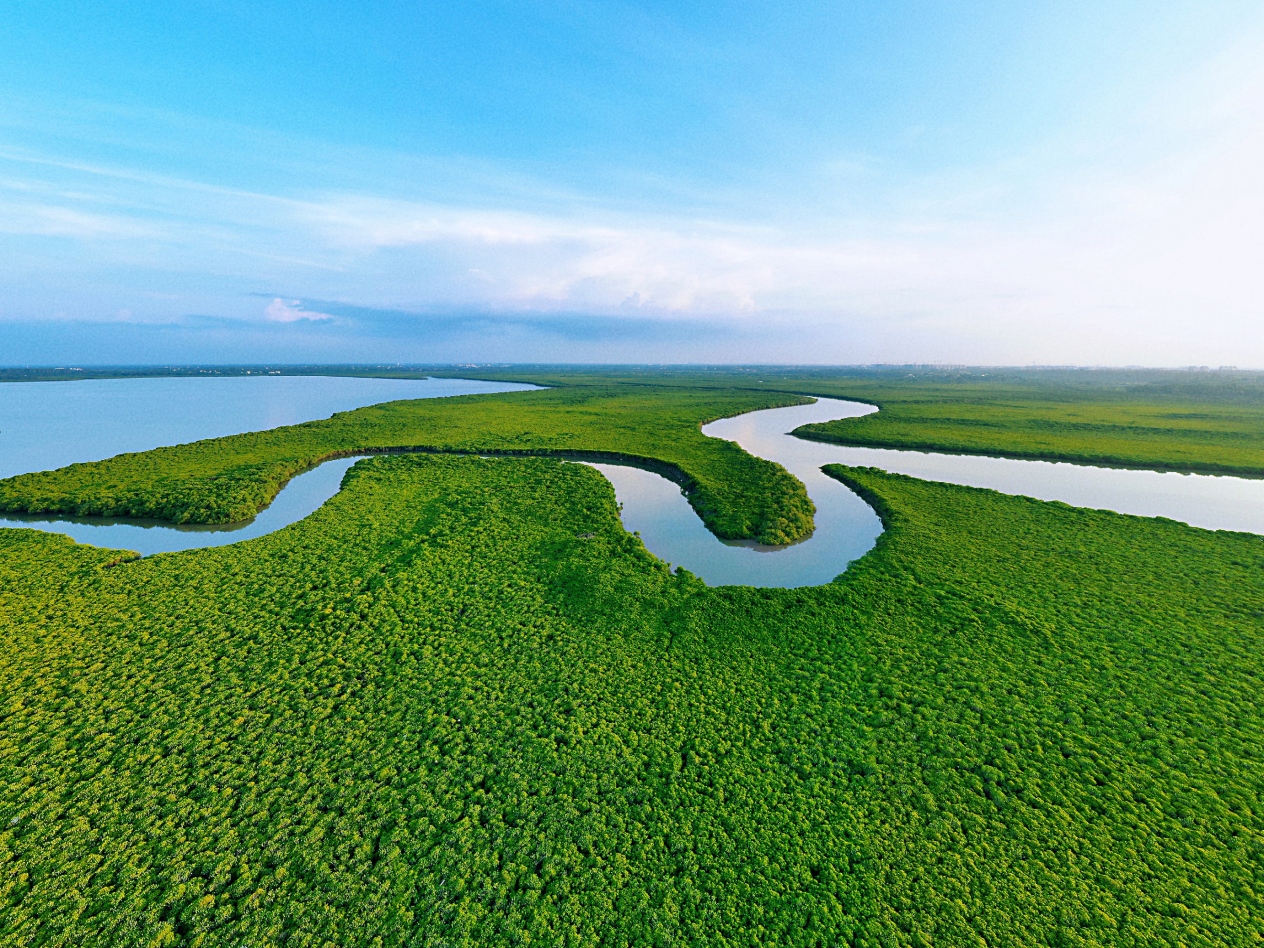
Aerial view over mangrove forests and estuaries at Dongzhaigang National Nature Reserve in northeastern Hainan Province, China. Photographer: Erhui Feng.

The experiment was conducted at the Dongzhaigang National Nature Reserve (Figure 1; 110°32′–110°37′ E and 19°51′–20°01′N) in the northeastern Hainan Province, China. The reserve is the earliest established mangrove reserve in China, covering 3337.6 ha. This area is characterized by a semi‐enclosed estuary with a muddy bottom, nourished by four small rivers. It experiences semidiurnal tidal cycles, averaging a tidal range of 1.6–1.8 m. The climate is characterized as a tropical maritime monsoon with an average annual rainfall of 1676.4 mm and a mean annual temperature ranging from 23.3°C to 23.8°C (Li et al. 2016). A total of 35 species of mangrove plants have been documented across 25 genera and 18 families. This included 24 species of true mangroves, which belong to 14 genera and 10 families, as well as 11 species of minor mangroves, categorized under 11 genera and 8 families (Jiang et al. 2023). Deforestation ceased in 1986 when the bay was declared a national nature reserve. The dominant mangrove species are *Avicennia marina*, *Aegiceras corniculatum*, *Bruguiera sexangula*, *Ceriops tagal*, and *Rhizophora stylosa*.

### 2.2 Field Survey

The field survey was conducted during the peak of the rainy season. We selected five tree species and five shrub species for this study based on previous field investigations and literature research (Bai et al. 2021; Yu et al. 2023). Among our sampled species, five were located in the low intertidal zone and five were in the high intertidal zone (Table 1). Four plots (10 m × 10 m, > 1 km apart) were established for each species. The height and diameter at breast height (DBH; 1.3 m for tree) or basal height (0.2 m for shrubs) of each individual were recorded. For each species, we collected 30 current‐season, fully expanded, light‐exposed mature and healthy green leaves from three adult individuals per plot and mixed them as a composite sample. All leaves were placed in plastic bags and immediately stored in a cooler with ice. Subsequently, we transported the samples to the laboratory for the measurements of leaf structural traits.

**TABLE 1:** List of species, genera, families, intertidal gradients, growth forms, plant height, and diameter at breast height or basal height (DBH, means ± SE) in the study.

Species	Genera	Families	Intertidal zone	Growth form	Plant height (m)	DBH (cm)
*Avicennia marina*	Avicennia	Acanthaceae	Low	Shrub	1.86 ± 0.26	5.37 ± 0.74
*Kandelia candel*	Kandelia	Rhizophoraceae	Low	Shrub	2.39 ± 0.13	5.83 ± 0.87
*Aegiceras corniculatum*	Aegiceras	Primulaceae	Low	Shrub	1.32 ± 0.40	4.48 ± 0.82
*Sonneratia caseolaris*	Sonneratia	Lythraceae	Low	Tree	8.83 ± 1.66	16.03 ± 2.36
*Sonneratia apetala*	Sonneratia	Lythraceae	Low	Tree	7.92 ± 1.57	14.40 ± 3.84
*Ceriops tagal*	Ceriops	Rhizophoraceae	High	Shrub	1.72 ± 0.14	4.77 ± 0.41
*Lumnitzera racemosa*	Lumnitzera	Combretaceae	High	Shrub	2.13 ± 0.15	6.56 ± 1.26
*Rhizophora stylosa*	Rhizophora	Rhizophoraceae	High	Tree	3.25 ± 0.30	4.55 ± 0.24
*Bruguiera sexangula*	Bruguiera	Rhizophoraceae	High	Tree	3.12 ± 0.82	6.20 ± 2.34
*Bruguiera gymnorrhiza*	Bruguiera	Rhizophoraceae	High	Tree	3.25 ± 0.34	5.34 ± 0.53

### 2.3 Leaf Structural Traits

The fresh leaf chlorophyll content (LCC) was estimated with a portable optical chlorophyll meter (SPAD‐502, Konika‐Minolta Inc., Tokyo, Japan). The LA was determined with an LA meter (LI‐3000c, Lincoln, Nebraska, USA). Additionally, LT was measured using a digital micrometer (Digimatic Micrometer, Mitutoyo, Japan). This measurement was derived from the average of three randomly selected positions on each leaf, deliberately avoiding the prominent veins to ensure accuracy on flat leaf surfaces. Leaf fresh mass (LFM) was weighted using a balance (0.0001 g, Meilen, Meifu Electronics Co. Ltd., Shenzhen, China). Following the rehydration procedure, the leaves were carefully dabbed with tissue paper to eliminate any residual surface moisture prior to measuring the leaf saturated mass (LSM). Samples were subsequently oven‐dried to a constant mass at 65°C for at least 48 h and then weighed to obtain the leaf dry mass (LDM).

Leaf volume (LV) was estimated using LA multiplied by LT. The LMA, the reciprocal of the SLA, was calculated using the LDM divided by the LA. Leaf density (LD) was calculated by dividing LMA by LT. The LDMC was calculated as the ratio of LDM to LSM. Finally, water saturation deficit (WSD), a critical parameter widely utilized for assessing plant tolerance to temporary water shortages, was calculated as follows (Lal et al. 2009):

$$\mathrm{WSD}\left(\%\right)=\left(\mathrm{LSM}-\mathrm{LFM}\right)/\left(\mathrm{LSM}-\mathrm{LDM}\right)\times 100\%$$

### 2.4 Statistical Analyses

All the statistical analyses were conducted using R version 4.3.0. Normality, homoscedasticity, and model fit were assessed using residual plots, Shapiro–Wilk test, and Levene's test. First, we conducted two‐way analysis of variance (ANOVA) using general linear model procedures to test for the main effects of intertidal gradients and growth forms and their interactions on leaf traits. When the effects of treatments were significant, mean comparisons were performed using the “*emmeans*” package (Song et al. 2023; Cheng et al. 2024). Second, in order to determine the respective influences of the interspecific and intraspecific variabilities on trait characteristics, we conducted a mixed‐effects model where an individual (id) was specified as a fixed effect and species was treated as a random effect (Luiz et al. 2022). Subsequently, we employed the r.squaredGLMM function from the “*MuMIn*” package to calculate the marginal *R* ^2^ (fixed‐effects only) and conditional *R* ^2^ (fixed and random effects) (Nakagawa and Schielzeth 2013). The discrepancy between these two *R* ^2^ values elucidates the extent to which the random effects contribute to the model. Third, phylogenetic signals of all traits were calculated with Blomberg's *K* statistic (Blomberg, Garland, and Ives 2003) using the “*picante*” package (Bergmann et al. 2020). This test compares the variance of the phylogenetically independent contrast of the study trait against those obtained with data randomly reshuffled in the phylogeny. A *K* value close to 1 indicates a significant phylogenetic effect, while a value close to 0 suggests no phylogenetic signal. In this study, the *K* values were < 1, and the corresponding *p* values were > 0.05 for all traits, suggesting a lack of phylogenetic conservatism (Table S1). To investigate multivariate trait relationships, we performed principal component analysis (PCA) on all 11 leaf traits and plant sizes using the “*vegan*” package. Finally, we used simple regression analyses to examine the effects of plant height and DBH on LCC, LD, LDMC, WSD, and LMA and used general linear models to test the difference in regression slopes between intertidal zones and growth forms.

## 3 Results

Leaf structural traits varied significantly among the 10 sampled mangrove species (Table 2 and data table available in the Supporting Information). The most variable traits were LV, LFM, LSM, and LDM, with a coefficient of variation (CV) of approximately 60%. In contrast, LCC, LT, LDMC, LD, and LMA were the least variable, with CVs < 30%. LA and WSD were moderately variable (CV = 48.81% and 42.00%, respectively). We found that the variability of leaf trait values was mostly explained by interspecific variations, rather than variations within individual species (intraspecific variations) (Figure S1).

**TABLE 2:** Leaf structural traits (units), their mean values, ranges, and coefficients of variation (CVs) in this study.

Leaf structural trait	Abbreviation	Mean	Min	Max	CV (%)
Leaf chlorophyll content (SPAD)	LCC	64.91	43.28	86.33	16.62
Leaf area (cm^2^)	LA	18.44	7.62	41.36	48.81
Leaf thickness (mm)	LT	0.55	0.36	0.82	23.64
Leaf volume (cm^3^)	LV	1.03	0.34	2.76	61.17
Leaf fresh mass (g)	LFM	1.05	0.36	2.62	60.00
Leaf saturated mass (g)	LSM	1.18	0.43	2.90	59.32
Leaf dry mass (g)	LDM	0.30	0.10	0.75	63.33
Leaf dry mass content (%)	LDMC	25.38	14.42	34.34	16.58
Leaf density (g cm^−3^)	LD	0.29	0.14	0.40	20.69
Water saturation deficit (%)	WSD	14.00	5.14	23.40	42.00
Leaf mass per area (g m^−2^)	LMA	159.39	89.26	242.71	22.84

**FIGURE 2:**
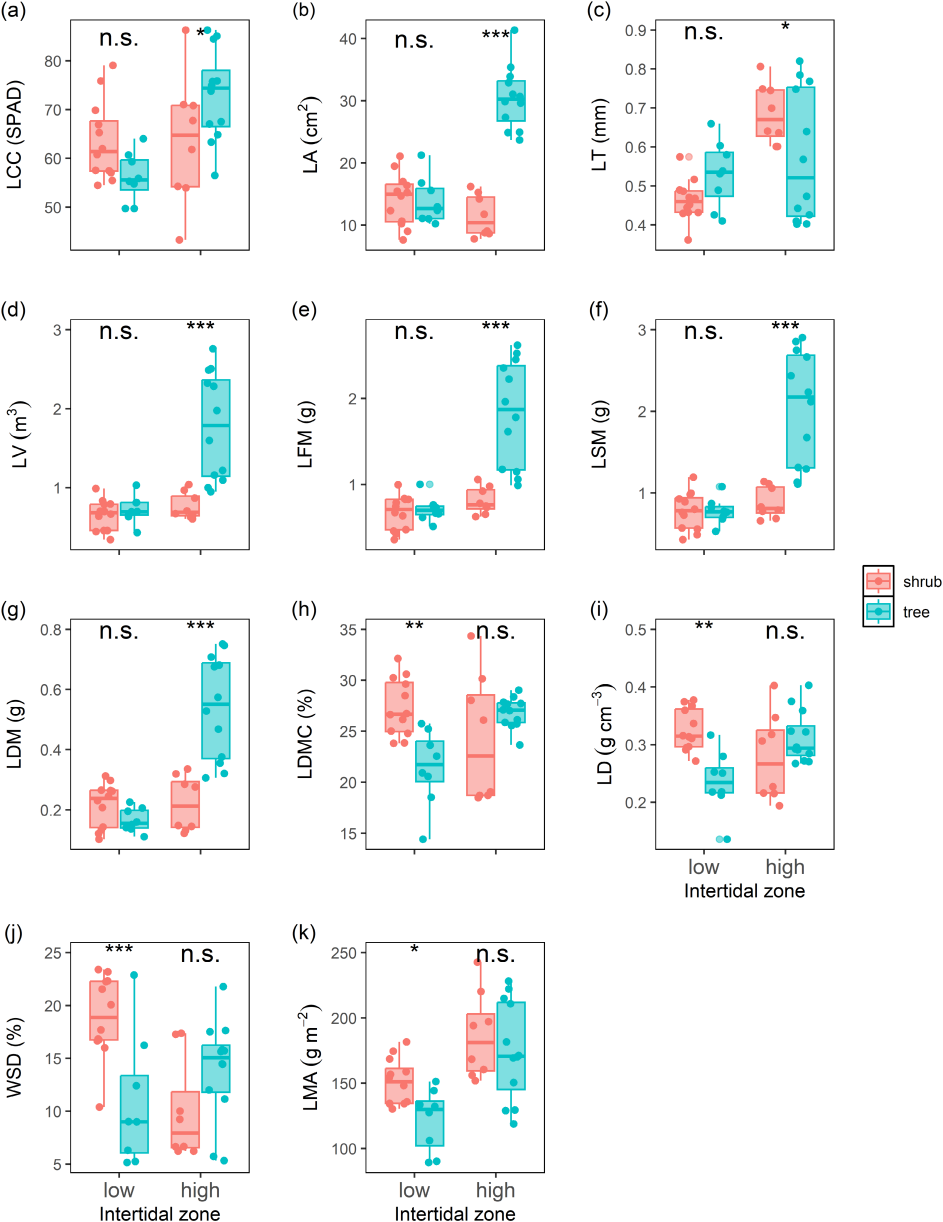
Leaf structural traits of shrubs and trees within low‐ and high‐elevation intertidal zones. Trait abbreviations are provided in Table 2. Statistically significant differences between shrubs and trees are shown with asterisks (***p < 0.001, **p < 0.01, *p < 0.05). n.s. = not significant.

The LCC, LA, LT, LV, LFM, LSM, LDM, and LMA increased significantly, and the WSD decreased from the low to high intertidal zones (Table 3 and Figure 2, *p* < 0.05). However, no significant differences between intertidal gradients were found for LDMC and LD. Compared with shrubs, trees had greater LA, LV, LFM, LSM, and LDM and lower LMA (Table 3 and Figure 2, *p* < 0.05), while the main effect of growth form had no significant effect on the other traits. Additionally, we found significant interactive effects between growth forms and intertidal gradients on all traits, except for LMA. Trees in high intertidal zones had greater LCC, LA, LV, LFM, LSM, and LDM and lower LT than shrubs did (Table 3 and Figure 2, *p* < 0.05), while differences in these traits between the growth types were not apparent in low intertidal zones. The LDMC, LD, and WSD were higher for shrubs than for trees only in low intertidal zones (Table 3 and Figure 2, *p* < 0.05), but these differences were not significant in high intertidal zones.

**TABLE 3:** Effects of intertidal zone gradient, growth form, and their interactions on leaf traits. The trait abbreviations are shown in Table 2.

Leaf trait	Intertidal gradient	Growth form	Intertidal gradient × Growth form
	*F*, *P*	*F*, *P*	*F*, *P*
LCC	**8.71** **	0.11	**7.67** **
LA	**43.31** ***	**47.03** ***	**48.97** ***
LT	**13.61** ***	0.39	**6.26** *
LV	**29.58** ***	**17.24** ***	**13.04** ***
LFM	**39.26** ***	**19.42** ***	**16.58** ***
LSM	**35.62** ***	**18.01** ***	**17.94** ***
LDM	**37.50** ***	**14.01** ***	**26.19** ***
LDMC	0.45	1.92	**12.89** ***
LD	0.33	2.77	**13.98** ***
WSD	**4.71** *	1.77	**15.07** ***
LMA	**18.67** ***	**4.66** *	0.85

*Note:* Significant results (*p* < 0.05) are shown in bold.

* *p* < 0.05.

** *p* < 0.01.

*** *p* < 0.001.

**FIGURE 3:**
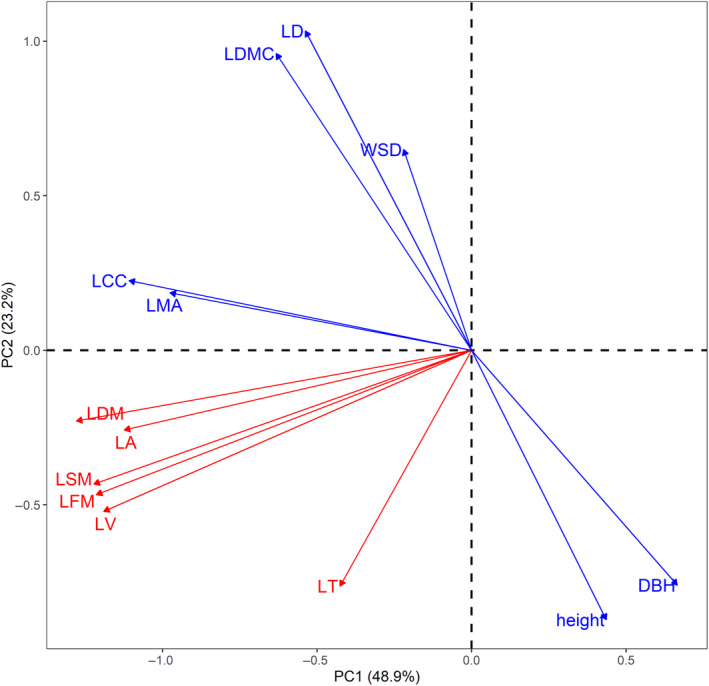
Principal component analysis (PCA) conducted on 11 leaf traits and plant height and diameter at breast height or basal height (DBH) among 10 mangrove species in Dongzhaigang, China. Trait abbreviations are provided in Table 2.

The PCA results showed that Axis 1 and Axis 2 explained 48.9% and 23.2% of the total variance, respectively (Figure 3). Two independent dimensions of trait variation stood out within this plane. One dimension (upper left to lower right in Figure 3) ran from short and small DBH species with “conservative” leaves (high LMA, LDMC, and LD) to tall and large DBH species with “acquisitive” leaves (low LMA, LDMC, and LD). The other ran from large LA species tending to have thick and heavy leaves to small LA species tending to have thin and light leaves (lower left to upper right in Figure 3).

**FIGURE 4:**
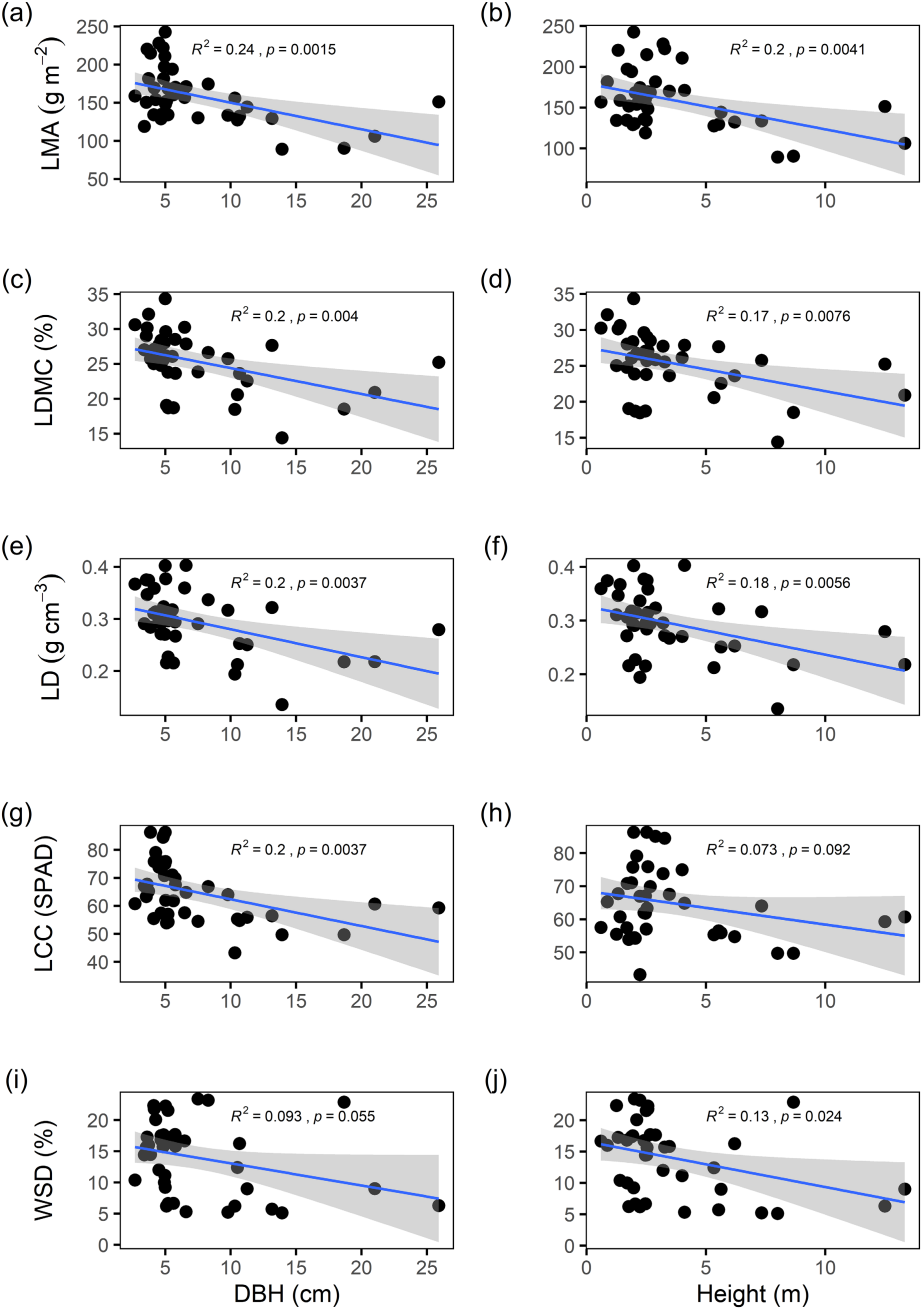
Relationships between leaf traits and plant size. The coefficients of determination (*R* ^2^) and *p* values are shown in each panel. The trait abbreviations are provided in Table 2.

Among all morphological and physiological characteristics, LMA, LDMC, LD, LCC, and WSD were negatively correlated with plant height and DBH (Figure 4). Similar patterns of LMA, LDMC, LD, LCC, and WSD in relation to plant height and DBH were found when intertidal zones and growth forms were analyzed individually, with LMA, LDMC, LD, LCC, and WSD decreasing with plant height and DBH (Figures S2 and S3). When regression slopes between different growth forms and intertidal gradients were tested, only LCC between high and low tides responded differently to plant DBH (*p* < 0.05, Table S2).

## 4 Discussion

We examined the impacts of plant form and intertidal gradient on leaf structural traits and analyzed the relationships between leaf structural traits and plant size among 10 dominant mangrove species. Our findings indicate that most leaf traits are significantly affected by growth form, intertidal gradient, and their interactions, consistent with our first alternative hypothesis. We also found a negative relationship between leaf economic traits (LMA, LDMC, and LD) and plant size, consistent with our second alternative hypothesis.

### 4.1 Growth Forms and Intertidal Gradients Affect Leaf Traits

We found that most leaf structural traits of mangrove species differed significantly along intertidal gradients. Specifically, the LCC, LA, LT, LV, LFM, LSM, LDM, and LMA increased significantly, and the WSD decreased with elevation. These results differed from those of a previous study of mangrove plants along an intertidal gradient in mangrove wetlands in Hainan, China, which reported that LMA and LT decreased significantly from low to high intertidal zones (Yu et al. 2023). The observed differences are likely due to species‐specific adaptive mechanisms of mangroves to the challenging environmental conditions characterized by high salinity, low oxygen levels, and acidity in intertidal zones (Friess et al. 2019). For example, del Campo et al. (2022) demonstrated that the LDMC, LT, and LMA of five mangrove species (*Laguncularia racemose*, *Rizhophora mangle*, *Prosopis julifora*, *Guazuma ulmifolia*, and *Ficus insipida*) increase with increasing salinity and decreasing pH, indicating greater conservation of plants in highly stressed soils (Wright et al. 2004; Díaz et al. 2016). However, species such as *A. marina* have developed specialized salt glands on their leaves that excrete excess salt, thereby preventing toxic salt accumulation (Natarajan et al. 2021). Furthermore, mangroves exhibit stomatal regulation as a response to tidal fluctuations, closing their stomata during high tide to reduce water loss and salt uptake and reopening them during low tide to promote gas exchange (Qie et al. 2024). Yu et al. (2023) concentrated their research on three species—*Kandelia obovata*, *B. sexangula*, and *Pongamia pinnata*—whereas our study expanded the spectrum to encompass 10 distinct species. This broadened taxonomic scope may account for the divergent findings observed in our research.

Our findings that trees had greater LA, LV, LFM, LSM, and LDM and lower LMA than shrubs did were partially consistent with a study by Wang et al. (2019), who reported that LA, LDM, and LMA were greater in trees than in shrubs. LA reflects a plant's light capture potential (Strauss et al. 2020). Tree canopies are typically exposed to high irradiance, while understory shrubs may face constraints in terms of the availability of light resources (Kenzo et al. 2015; He and Yan 2018). Therefore, higher LA in trees may be an adaptation to high light intensity to maintain greater photosynthetic capacity and productivity. Moreover, higher LV, LFM, LSM, and LDM can enhance photosynthetic capacity under high irradiance. This is achieved by increasing the nitrogen content and expanding the photosynthetic machinery volume per unit LA (Oguchi, Hikosaka, and Hirose 2005; Liu et al. 2019), which may explain the greater LV, LFM, LSM, and LDM in trees. LMAs are the primary driving factors of drought tolerance (Fletcher et al. 2018). Our findings indicate that mangrove shrubs may experience more limited access to water resources than trees, as evidenced by the greater WSD observed for shrubs. Additionally, a high LMA suggests a reduction in the intercellular space and increased resistance to gas diffusion within the mesophyll (Peguero‐Pina et al. 2017). This characteristic could be advantageous because it enhances plant tolerance to cell collapse, a consequence of drought stress (Bussotti and Pollastrini 2015; Evans 2021). The diffusion resistance of shrubs with a high LMA may increase or decrease leaf transpiration. Hence, determining the variation in leaf traits between different growth forms is essential for elucidating the mechanisms that underpin the ecological strategies of plant species. These strategies are crucial for the successful adaptation and occupancy of diverse habitats (Wang et al. 2022; Islam et al. 2024).

Exploring the effects of intertidal gradients on leaf functional traits between plant growth forms is helpful for understanding species diversity maintenance in forests (del Campo et al. 2022; Yu et al. 2023). Our study revealed that the gradients had a significant impact on leaf structural traits (LA, LV, LFM, LSM, and LDM) of different growth forms. Leaf functional trait (SLA, LT, and LDMC) variation with growth form effectively reflects a plant's adaptation strategy, which shapes differences in their demand and utilization of resources such as light, precipitation, temperature, and nutrients (Islam et al. 2024). Thus, leaf traits differ among growth forms in response to intertidal elevation gradients with changes in moisture, temperature, salinity, and wave energy (Wang et al. 2019). Our study highlights the different effects of intertidal gradients on leaf traits between different plant growth forms. This enhanced understanding is expected to deepen our insight into plant adaptive strategies and the evolutionary dynamics of plant traits as they adapt to the mosaic of environmental conditions. In addition, our findings reveal a major mechanism maintaining plant diversity in mangrove forests.

### 4.2 A Trade‐Off Between Leaf Economics and Plant Size

Despite a wealth of research into trait relationships in terrestrial plants (Prieto et al. 2018; Simpson et al. 2020; Zhou, Cieraad, and van Bodegom 2022), our comprehension of leaf trait relationships in mangroves remains comparatively limited, primarily due to the scarcity of research in this area. Our study revealed that large mangrove species (tall and large DBHs) tended to have low leaf structural investment (low LMA, LD, and LDMC). This is contrary to several studies that have shown positive relationships between LMA and tree size across developmental stages for conspecific individuals (Nouvellon et al. 2010; He and Yan 2018; Liu et al. 2020). There are three possible interpretations for this difference. First, the size and leaf traits may have different drivers. A global analysis indicates that plant size varies with latitude due to water and energy constraints, while economic traits are influenced by climate and soil fertility interactions (Joswig et al. 2022). These drivers may co‐occur among species while being decoupled within species (Anderegg et al. 2018; Zhou, Cieraad, and van Bodegom 2022). Thus, the negative size–trait relationships found across species disappeared when the analysis focused on intraspecific patterns. Second, decreases in LMA are often interpreted as a strategic adaptation by plants to enhance their light‐harvesting efficiency (del Campo et al. 2022). The canopies of taller plants experience greater solar irradiance compared to those of their shorter counterparts (Maynard et al. 2022). These differences in the light exposure of plant crowns may be instrumental in the observed variability in LMA. Finally, decreases in LMA may result from decreases in leaf water stress (e.g., WSD) with increasing plant size (Cao et al. 2023). Our study underscores the importance of elucidating the underlying mechanisms behind trait–trait relationships, both within and among species. These mechanisms are essential for deepening our insight into the intricate adaptive strategies employed by plants in their quest for survival and success.

Our results were inconsistent with previous research on global plant size–trait relationships (Díaz et al. 2016; Joswig et al. 2022; Maynard et al. 2022). A previous study pinpointed a crucial collection of functional traits that summarize the spectrum of forms and functions within the plant kingdom, with leaf economics (e.g., LMA) and plant size (e.g., tree height) being the two dominant dimensions underpinning life‐history strategies (Maynard et al. 2022). The distinct orthogonality of these two axes implies that they are shaped by different environmental drivers. For example, a comprehensive global assessment examining 17 traits across an extensive sample of over 20,000 species demonstrated that variations in size‐related traits are strongly associated with latitudinal gradients, which are indicative of constraints on water or energy availability (Joswig et al. 2022). In contrast, economic traits show a near‐exclusive response to soil conditions, highlighting the unique influence of soil factors on these characteristics. Therefore, these interspecific size–trait relationships are confounded by environmental drivers at a global scale.

Consistent with previous research (Kenzo et al. 2006; Louis et al. 2012), we found that LCC significantly decreased with plant height. High LCC leaves are related to low dark respiration rates and light compensation points, permitting better acclimation to poor light for small trees and shrubs (Guimarães, dos Santos, and Ferreira 2022). Negative correlations between LCC and plant height indicate that a high LCC contributes to light‐harvesting efficiency at low irradiances.

Despite substantial differences in leaf traits among growth forms and intertidal gradients, negative relationships between leaf traits (LMA, LDMC, LD, LCC, and WSD) and plant size (height and DBH) were detected within different growth forms and intertidal zones. Our results disagree with the findings of Li et al. (2021), who utilized leaf trait networks derived from global data to assert that the interdependence of leaf economic traits was more pronounced in shrubs than in trees. Plants in environments with limited resource availability are likely subjected to more intense selective pressures, leading to a tighter correlation between traits to ensure efficient resource acquisition and utilization (Flores‐Moreno et al. 2019; Liu et al. 2019). For instance, leaf economic and hydraulic traits are found to be independent in humid regions (Li et al. 2015) but exhibit strong coupling in arid regions (Yin et al. 2018). In comparison to terrestrial plants, mangroves may face more constrained availability of water resources since water uptake under saline conditions is energetically expensive (Santini et al. 2015). Consequently, mangroves adopt a cost‐effective strategy that promotes a strong correlation between leaf traits and plant size across growth forms and intertidal gradients, facilitating efficient functioning.

Plant leaves are subject to strong selective pressures to optimize their C economics (Reich 2014; Deans et al. 2020). For example, species in arid and semiarid regions typically have high LMA, indicating higher leaf construction costs from accumulating costly C‐based compounds like lignin or lipids, while those with low LMA tend to accumulate less expensive compounds such as water or minerals (Wright et al. 2004). Understanding the economic principles of C use in mangrove leaves is essential for deciphering the C cycle in coastal ecosystems, as it links plant size (height and DBH) with the C costs of leaf construction and turnover (LMA, LDMC, and LD).

## 5 Conclusions

We examined the responses of leaf structural traits to growth form and intertidal gradient as well as the associations between leaf traits and plant size across mangrove species in Dongzhaigang, China. Our findings revealed that leaf traits differed significantly among growth forms in response to intertidal gradients. These findings contribute to a deeper understanding of plant adaptive strategies and trait evolution in response to diverse environmental conditions. Negative relationships between leaf C economics traits (LMA, LDMC, and LD) and plant size (height and DBH) were found for each growth form and intertidal zone, as well as for the pooled dataset. Achieving a thorough comprehension of these relationships is critical for advancing plant functional ecology and refining global C cycle models, which are sensitive to atmospheric CO_2_ fluctuations and climate change. It is imperative to emphasize that additional studies are warranted to elucidate intraspecific trait variability at local scales. Such research would significantly enhance our understanding of community assembly dynamics and the mechanisms by which plant communities influence ecosystem processes (Kumordzi et al. 2014). Exploring the variability of intraspecific traits at local scales, both among communities and across environmental gradients, represents a promising and intriguing pathway for future scientific investigations.

## Author Contributions

**Dalong Jiang:** conceptualization (lead), formal analysis (lead), funding acquisition (lead), project administration (lead), writing – original draft (lead). **Tao Nie:** data curation (equal), investigation (equal), methodology (equal). **Qiuyu He:** data curation (equal), investigation (equal), methodology (equal). **Jing Yan:** data curation (equal), investigation (equal), methodology (equal). **Erhui Feng:** investigation (equal), resources (equal). **Qing Ye:** conceptualization (equal), supervision (lead), writing – review and editing (lead).

## Conflicts of Interest

The authors declare no conflicts of interest.

## Supporting information


**Figure S1.** Decomposition of the variability of leaf trait values into interspecific variations, intraspecific variations, and unexplained.
**Figure S2**. Relationships between leaf traits and plant size, fitted by regression for shrubs and trees. The coefficients of determination (*R* ^2^) and *p* are shown in each panel. The trait abbreviations are provided in Table S1.
**Figure S3**. Relationships between leaf traits and plant size, fitted by regression for low‐ and high‐elevation intertidal zones. The coefficients of determination (*R* ^2^) and *p* are shown in each panel. The trait abbreviations are provided in Table S1.
**Table S1**. Blomberg’s *K* for each leaf trait.
**Table S2**. Results of analysis of covariance (ANCOVA) with leaf traits (LCC, LDMC, LD, WSD, and LMA) as dependent variables, plant height and diameter at breast height or basal height (DBH) as covariates, and intertidal gradients and growth forms as the factors. Values in bold indicate significant effects (*p* < 0.05).

Data S1.

## Data Availability

Data and R code supporting this study are archived in Dryad Digital Repository: https://doi.org/10.5061/dryad.tmpg4f581.
