# Supplementary material for: A Trade‐Off Between Leaf Carbon Economics and Plant Size Among Mangrove Species in Dongzhaigang, China
Source: Ecol Evol. 2024 Nov 19;14(11):e70559. doi: 10.1002/ece3.70559 (PMC11576130; doi:10.1002/ece3.70559)
Supplement: Supplementary file 1 — Figure S1. Decomposition of the variability of leaf trait values into interspecific variations, intraspecific variations, and unexplained. Figure S2. Relationships between leaf traits and plant size, fitted by regression for shrubs and trees. The coefficients of determination (R 2) and p are shown in each panel. The trait abbreviations are provided in Table S1. Figure S3. Relationships between leaf traits and plant size, fitted by regression for low‐ and high‐elevation intertidal zones. The coefficients of determination (R 2) and p are shown in each panel. The trait abbreviations are provided in Table S1. Table S1. Blomberg’s K for each leaf trait. Table S2. Results of analysis of covariance (ANCOVA) with leaf traits (LCC, LDMC, LD, WSD, and LMA) as dependent variables, plant height and diameter at breast height or basal height (DBH) as covariates, and intertidal gradients and growth forms as the factors. Values in bold indicate significant effects (p < 0.05). [file ECE3-14-e70559-s002.docx]

**Appendix**

**Table S1** Blomberg’s K for each leaf trait.

| Leaf traits | K | *p* |
| --- | --- | --- |
| LCC | 0.10 | 0.26 |
| LA | 0.44 | 0.07 |
| LT | 0.02 | 0.62 |
| LV | 0.21 | 0.28 |
| LFM | 0.28 | 0.16 |
| LSM | 0.26 | 0.21 |
| LDM | 0.38 | 0.11 |
| LDMC | 0.03 | 0.56 |
| LD | 0.01 | 0.76 |
| WSD | 0.02 | 0.59 |
| LMA | 0.30 | 0.15 |
| Height | 0.03 | 0.62 |
| DBH | 0.30 | 0.35 |

**Note:** leaf chlorophyll content (SPAD, LCC), leaf area (cm^2^, LA), leaf thickness (mm, LT), leaf volume (cm^3^, LV), leaf fresh mass (g, LFM), leaf saturated mass (g, LSM), leaf dry mass (g, LDM), leaf dry mass content (%, LDMC), leaf density (g cm^−3^, LD), water saturation deficit (%, WSD), leaf mass per area (g m^−2^, LMA), plant height (m, Height), diameter at breast height or basal height (cm, DBH).

**Table S2** Results of analysis of covariance (ANCOVA) with leaf traits (LCC, LDMC, LD, WSD, and LMA) as dependent variables, plant height and diameter at breast height or basal height (DBH) as covariates, and intertidal gradients and growth forms as the factors. Values in bold indicate significant effects (*p* < 0.05).

|  | LCC  (SPAD) | LDMC  (%) | LD  (g cm^‒3^) | WSD  (%) | LMA  (g m^‒2^) |
| --- | --- | --- | --- | --- | --- |
|  | *F, P* | *F, P* | *F, P* | *F, P* | *F, P* |
| DBH | **10.68**** | **9.59**** | **9.56**** | **3.77*** | **11.14***** |
| Growth form | **5.71*** | <0.01 | 0.06 | 0.58 | 0.04 |
| DBH× Growth form | 0.79 | 2.83 | 1.85 | 0.06 | 0.04 |
| Height | **3.41*** | **8.16**** | **8.57**** | **5.26*** | **9.06**** |
| Growth form | **6.00*** | 0.43 | 0.16 | 0.02 | 0.76 |
| Height × Growth form | 1.33 | 2.65 | 1.63 | 0.20 | 0.04 |
| DBH | **11.44**** | **8.97**** | **9.19**** | **4.92*** | **14.57***** |
| Tidal level | **3.80*** | 0.28 | 0.42 | **9.84**** | **10.12**** |
| DBH × Tidal level | **5.74*** | <0.01 | 0.04 | 2.01 | 1.10 |
| Height | **3.24*** | **7.78**** | **8.66**** | **6.54*** | **11.71**** |
| Tidal level | **5.13*** | 0.06 | 0.16 | **8.99**** | **11.50**** |
| Height × Tidal level | 0.02 | 1.20 | 2.06 | 0.04 | 0.05 |

**Note:** ***: *p* < 0.001, **: *p* < 0.01, *: *p* < 0.05


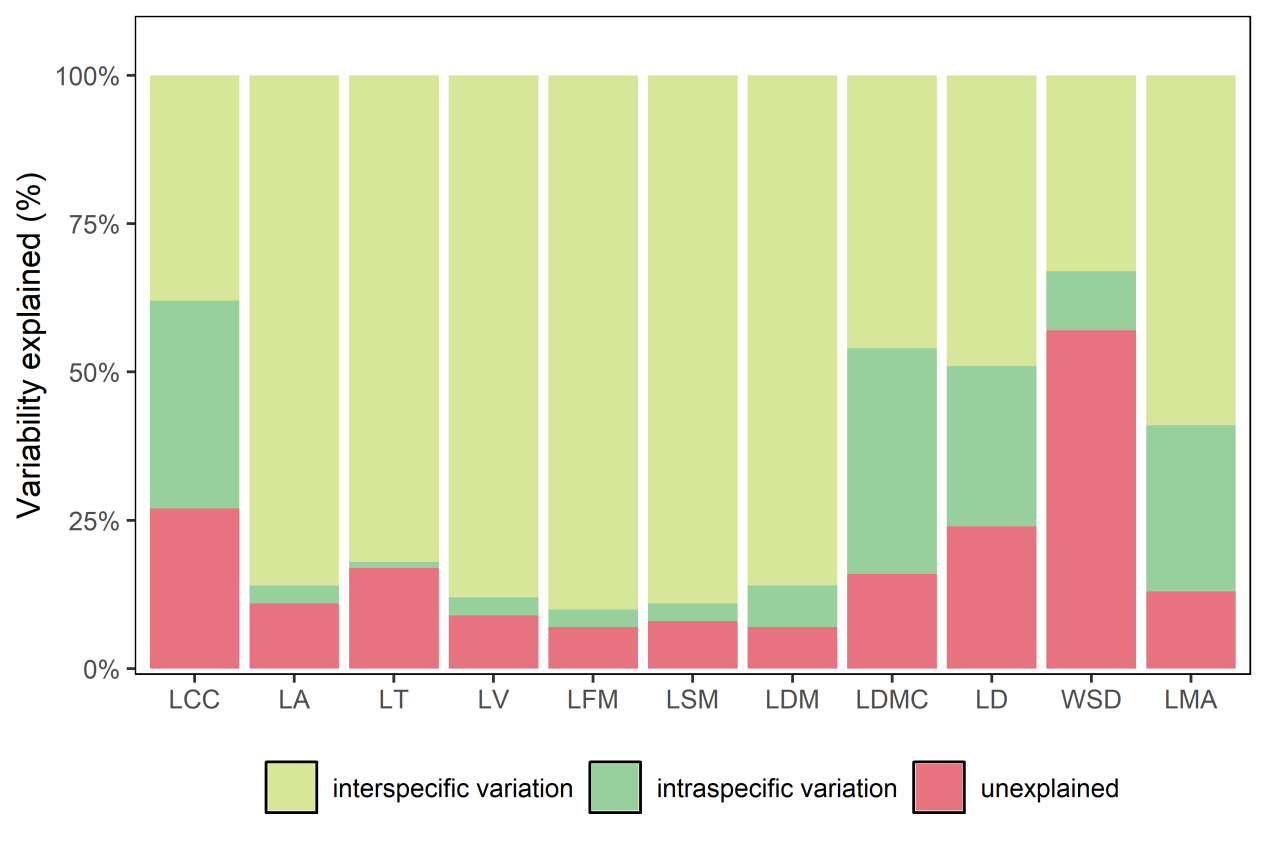


**Figure S1** Decomposition of variability of leaf trait values into interspecific variations, intraspecific variations, and unexplained.


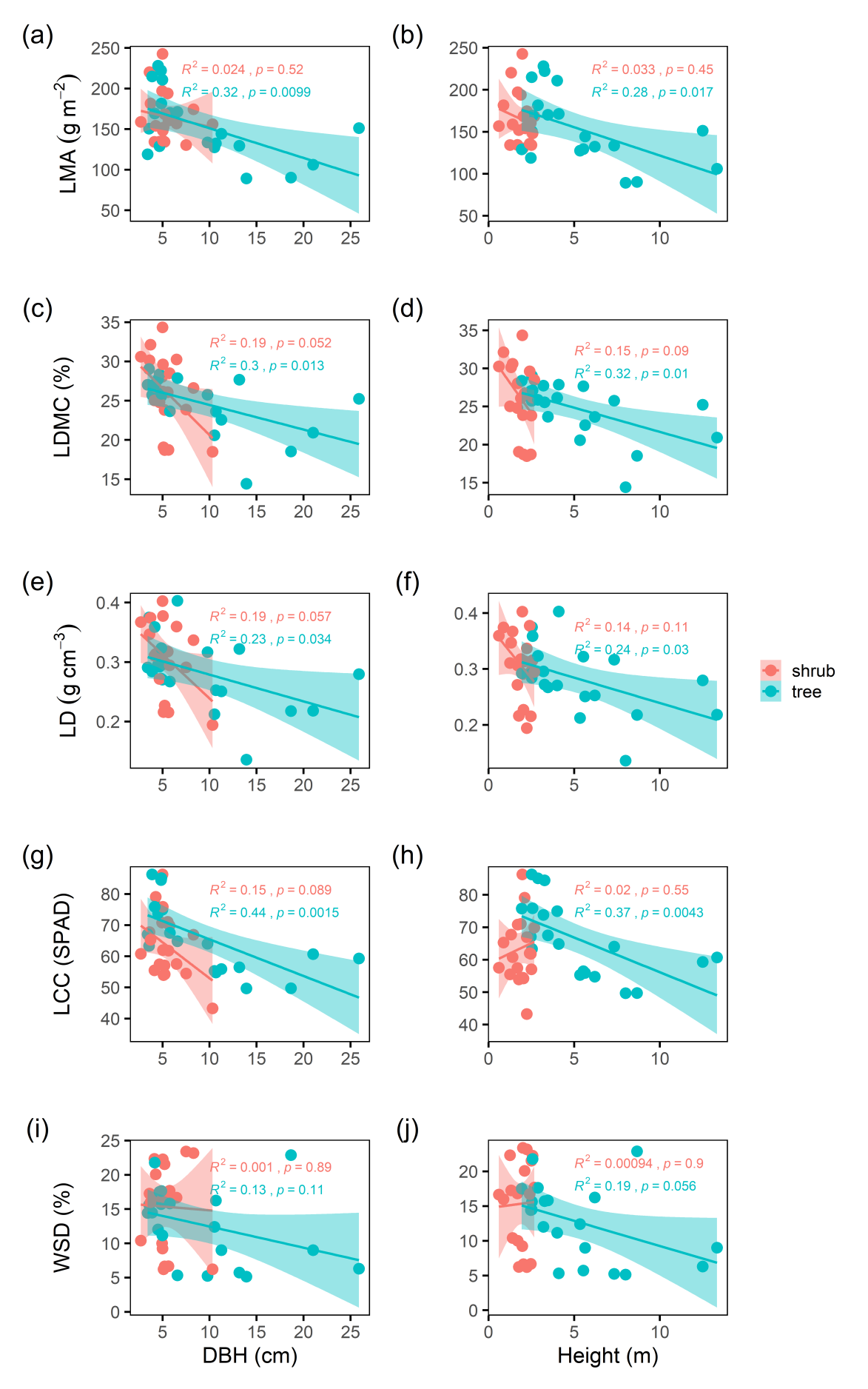


**Figure S2** Relationships between leaf traits and plant size, fitted by regression for shrubs and trees. The coefficients of determination (*R*^2^) and *p* are shown in each panel. The trait abbreviations are provided in Table S1.


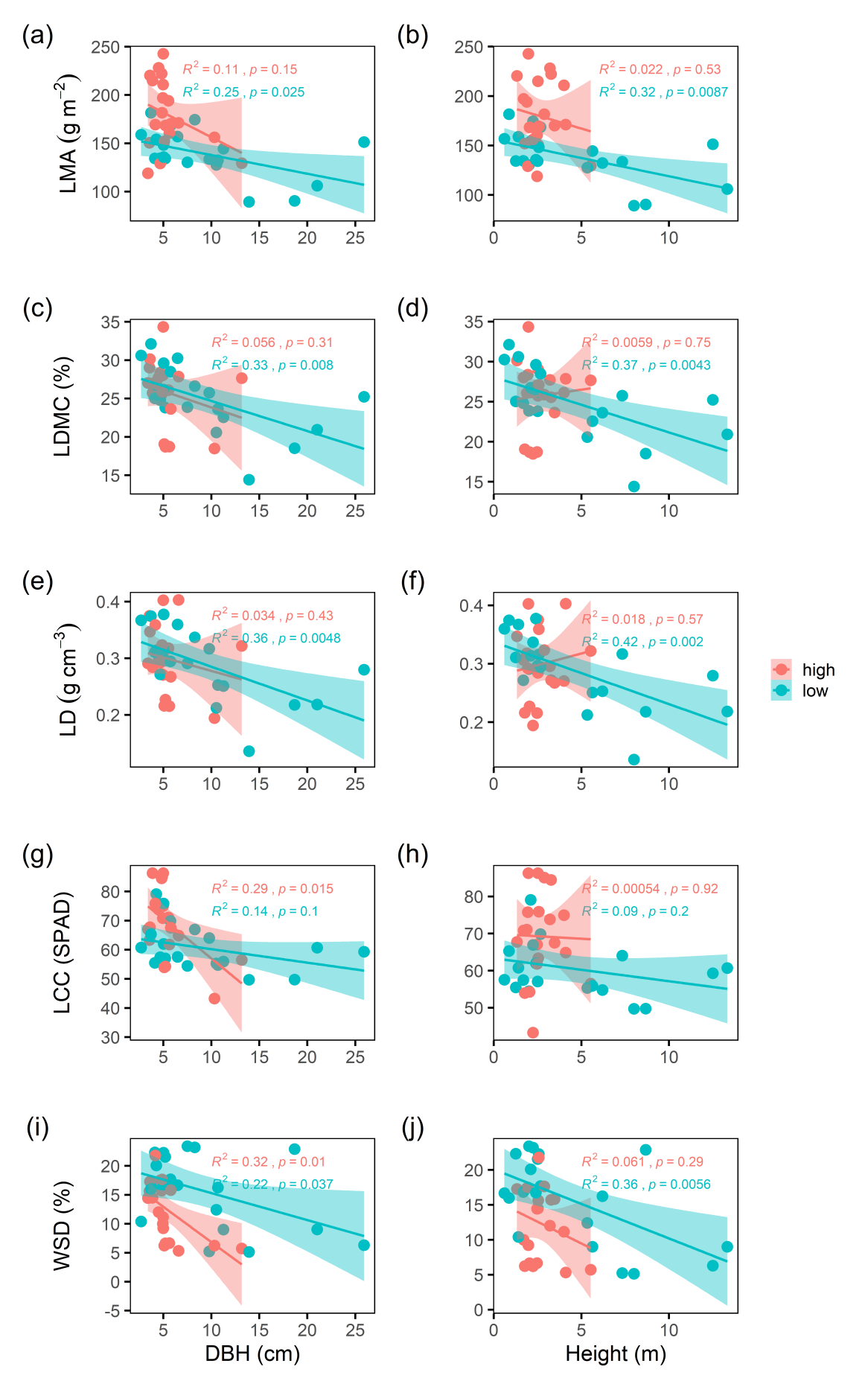


**Figure S3** Relationships between leaf traits and plant size, fitted by regression for low- and high-elevation intertidal zones. The coefficients of determination (*R*^2^) and *p* are shown in each panel. The trait abbreviations are provided in Table S1.
